# Endothelial Progenitor Cells for Ischemic Stroke: Update on Basic Research and Application

**DOI:** 10.1155/2017/2193432

**Published:** 2017-08-16

**Authors:** Shaohua Liao, Chunxia Luo, Bingzhen Cao, Huaiqiang Hu, Suxia Wang, Huili Yue, Lin Chen, Zhenhua Zhou

**Affiliations:** ^1^Department of Neurology, People's Liberation Army 152 Hospital, Pingdingshan, Henan 467000, China; ^2^Department of Neurology, Southwest Hospital, Third Military Medical University, Chongqing 400038, China; ^3^Department of Neurology, General Hospital of Jinan Military Region, Jinan, Shandong 250031, China

## Abstract

Ischemic stroke is one of the leading causes of human death and disability worldwide. So far, ultra-early thrombolytic therapy is the most effective treatment. However, most patients still live with varying degrees of neurological dysfunction due to its narrow therapeutic time window. It has been confirmed in many studies that endothelial progenitor cells (EPCs), as a kind of adult stem cells, can protect the neurovascular unit by repairing the vascular endothelium and its secretory function, which contribute to the recovery of neurological function after an ischemic stroke. This paper reviews the basic researches and clinical trials of EPCs especially in the field of ischemic stroke and addresses the combination of EPC application with new technologies, including neurovascular intervention, synthetic particles, cytokines, and EPC modification, with the aim of shedding some light on the application of EPCs in treating ischemic stroke in the future.

## 1. Introduction

In the world, stroke is the second cause of death and the leading cause of adult disability [1]. It is also the fifth cause of death and the leading cause of disabilities among American adults [2], of which 87% is ischemic stroke [3]. Hospitalized patients with ischemic stroke in China have a 3.3–5.2% mortality rate and a 34.5–37.1% death/disability rate 3 months after onset [4–6]. In the pathological process of ischemic stroke, the blood supply is interrupted after cerebral vascular occlusion, together with energy failure, acidosis, excitatory amino acid release, intracellular calcium overload, and generation of free radicals, which eventually lead to brain parenchymal damages composed of necrosis, apoptosis, and autophagy [7–11]. However, the treatment of ischemic stroke is still very limited. Clinical trials on neuroprotective drugs have not been successful [12], and the only FDA-approved treatment of acute stroke is to apply t-PA within 4.5 hours after onset. The emerging intravenous rt-PA thrombolysis prior to intravascular therapy in recent years requires that the femoral artery puncture be performed 120–212.5 minutes after the onset of symptoms [13]. As such, there are only about 2%–5% of stroke patients who meet the criteria for intravenous t-PA with or without bridging therapy due to its narrow therapeutic time window [14, 15]. Most patients still live with varying degrees of neurological dysfunctions. Therefore, a new effective treatment is badly needed to change this situation.

EPCs are regarded as immature endothelial cells which circulate in the peripheral blood. In 1997, Asahara et al. [16] isolated CD34 and Flk1-positive mononuclear cells from the peripheral blood, and these cells were named EPCs because of endothelial cell characteristics in culture medium. It is now believed that EPCs are precursor cells of mature vascular endothelial cells, which belong to stem cell populations with self-renewal capacity that can differentiate into mature endothelial cells (ECs). EPCs are confirmed to insert into the endothelium of newly formed vessels in the ischemic area, which play an important role in the process of endothelial repair and angiogenesis after injury. Studies also verify that EPCs have the potency of secreting a variety of cytokines and growth factors, which provide nutritional and antiapoptotic support for the circulating and resident EPCs and other cells (ECs, cardiomyocytes, neurons, neural stem cells, and so forth). Circulating human EPCs injected into nude mice after transient middle cerebral artery occlusion (tMCAO) can protect the neurovascular unit and contribute considerably to the recovery of neurological function [17], which has made itself an important candidate for stem cell therapy. In this review, we discuss the current development of EPC research in ischemic cerebrovascular diseases. In the first section of this review, we describe the basic research in the field of EPCs, including the effect on blood vessels and secreting function of EPCs. In the second part, the clinical application of EPCs is introduced, specially emphasizing the combination of EPC application with new technologies. This review is ended with the consideration of the safety of EPC application, which needs to be carefully concerned in future clinical trials.

## 2. Basic Research

### 2.1. Dynamic Changes of EPCs under Pathophysiological Conditions

Under physiological conditions, a small poll of hematopoietic stem cells (HSCs) in the bone marrow niche were differentiated and released into circulation, which are bone marrow-derived EPCs marked with KDR+, CD34+, and CD133+, and the level of EPCs in the peripheral circulation is low [18–20]. The supplementation of some food, such as onion peel, black raspberry, fish oil, and red wine, may be helpful in increasing the number of circulating EPCs [21–24]. Multiple factors (cytokines released by target tissue, growth factors, sex hormones, etc.) mobilize EPCs to migrate from the bone marrow stroma into the blood circulation. This process relies on the activation by endothelial nitric oxide synthase (eNOS). Upregulation of vascular endothelial growth factor (VEGF) may mobilize EPCs to migrate into the blood circulation [25], and the release of EPCs from the bone marrow may also be promoted by upregulating granulocyte colony-stimulating factor (G-CSF) [26, 27]. EPC level and G-CSF level are elevated after acute myocardial infarction [28]. Parathyroid hormone (PTH) can also facilitate bone marrow stem cell (BMSCs) and/or progenitor cell release into circulation [29, 30]. Under hypoxic or inflammatory conditions, endothelial cells (ECs) can upregulate the expression of stromal cell-derived factor-1*α* (SDF-1*α*) [31] and interact with EPCs that highly express C-X-C chemokine receptor type 4 (CXCR4) [32, 33], which not only promotes EPC mobilization from the bone marrow but also stimulates EPC recruitment and adherence to the ischemic regional vascular endothelium [20, 34, 35]. Nitric oxide (NO) and erythropoietin (EPO) are currently considered to be key factors for EPC mobilization. EPCs themselves can also promote the aggregation of more circulating EPCs by releasing VEGF and SDF-1*α* [36].

In the process of EPCs migrating to ischemic or damaged areas, CXCR4/SDF-1 plays an important role in directing EPCs to migrate to the damaged vascular endothelium [31, 37]. The binding of interleukin-6 (IL-6) and glycoprotein (gp80 or gp130) expressed by EPCs promotes the proliferation and migration of EPCs [38]. Some drugs, such as statins, can promote EPC migration and proliferation and reduce EPC apoptosis by activating the Akt/NOS pathway and upregulating matrix metalloproteinase-2 (MMP-2) and MMP-9 expression [39], which enhance EPC function.

In the ischemic area, EPC homing to the damaged vessels is considered as an essential step in the interaction with ECs of many cytokines and their receptors. The interaction of P-selectin expressed by platelet and P-selectin glycoprotein ligand-1 (PSGL-1) expressed by EPCs plays a key role in the process of EPC adherence to neovascularization [40, 41]. In addition, the interaction of *β*1/*β*2 integrins with the ligands, intercellular adhesion molecule-1 (ICAM-1) and vascular cell adhesion molecule-1 (VCAM-1), expressed in ischemic vessel endothelium, high-mobility group box 1 (HMGB1) and gpIIb-dependent platelet aggregates, and *α*4 integrins also participate in and promote EPC adhesion and homing [41–46].

The interaction of VEGF and EPCs is complicated and lies in many steps. In the process of dynamic change, VEGF is one of the critical factors and plays an essential role for EPCs. VEGF has effects on mobilization and migration of EPCs through the receptor KDR [47]. In hypoxia circumstance, HIF-1*α* is activated in the damaged tissue, which leads to increased levels of VEGF. Then, the VEGF prompts a migration of EPCs and hematopoietic cells [48], and the migratory effects have been documented by several studies [49, 50]. The protection of neurovascular unit of VEGF secreted by EPCs is illustrated in the “Secreting Function of EPCs” section.

For the dynamic changes in the function and number of EPCs under ischemic or inflammatory conditions [51], the use of microbeads and Q-dot-based nanoparticle is superior to conventional flow cytometry in analyzing the microvesicles released from EPCs. Other studies used Dex-DOTA-Gd3^+^ as a magnetic resonance imaging (MRI) contrast agent to observe the survival period of transplanted EPCs in the rat hind limb ischemic model [52] or used DiI-Ac-LDL staining or ^111^In-oxine radioactive markers to track transplanted EPCs [17, 53, 54]. These methods can be used to monitor or track EPCs transplanted in the body, providing evidence for EPC-based clinical or preclinical trials.

### 2.2. The Effect of EPCs on Blood Vessels

EPCs display three fundamental activities within the vascular systems, which include secretion, repairing endothelial damage, and formatting new blood vessels in ischemic tissues [18]. The secreting function of EPCs is mainly described in the next paragraph. In the process of atherosclerosis, focal arterial lesions contain cholesterol, fibrosis, and inflammatory cell infiltrates [55, 56], which substantially indicate the destruction of a balance between endothelial damage and repair. EPCs homing into the artery wall may assist to repair the endothelial injury [57], although the mechanisms involved are still unclear. In the ischemic or inflammation condition, the damaged tissue may release a variety of factors and induce the mobilization of EPCs from the bone marrow to the peripheral blood [58]. Bone marrow-derived EPCs can home to the neovascularization site, proliferating and differentiating into ECs [59, 60] and participating in angiogenesis. The transplanted EPCs may also appear in the newly formed vascular endothelium of the ischemic site, participating in postischemic angiogenesis [16, 61]. It has been demonstrated that MMP9 plays a key role in poststroke EPC-induced angiogenesis [62]. Some factors including VEGF, SDF-1, platelet-derived growth factor (PDGF), and microparticles secreted by EPCs can stimulate tip and stalk cells [63], to promote angiogenesis and local EC proliferation and migration [64]. EPCs can also differentiate into ECs, replacing or directly integrating with the damaged endothelial layer [65–68] to repair the vascular endothelium. However, it is also argued that circulating EPCs may not directly replenish ECs, but activate resident ECs [69] by secreting VEGF, hepatocyte growth factor (HGF), and other factors, or releasing microvesicles from the cell membrane to transmit mRNA to ECs that promote EC proliferation, form microtubules, and reduce apoptosis [70]. EPCs also contribute to the recovery of vascular ECs by secreting exosomes, a nanoscale vesicle encapsulated by lipid membrane structures [71]. This new approach may play a dominant role in the working mechanism of EPCs.

### 2.3. Secreting Function of EPCs

The neurovascular unit is a complex network of interactions, including neurons, astrocytes, microglias, microvascular ECs, and pericytes [72]. EPCs interact with the neurovascular unit by secreting multiple factors [36, 73–75]. Moreover, EPCs secrete SDF-1*α* and VEGF, creating a microenvironment for neuronal survival and regeneration [76, 77].

Further studies have shown that EPCs secrete multiple growth factors such as VEGF, SDF-1*α*, and insulin-like growth factor-1 (IGF-1), which can not only recruit more circulating EPCs and maintain their survival but also protect the existing collateral circulation and neurovascular unit [78]. VEGF may also promote angiogenesis and stimulate the proliferation and migration of new neurons [79].

Wang et al. [80] confirmed that cocultured EPCs and neural progenitor cells (NPCs) may secrete VEGF and brain-derived neurotrophic factor (BDNF) and provide synergistic protection through activating the PI3K/Akt pathway and minimizing cerebral vascular EC ischemia/reperfusion injury. It has also been found that intravenous combined transplantation of bone marrow stromal cells (BMSCs) and EPCs contributes to the recovery of neurological function in the rat cerebral ischemia model, which may be achieved by high expression of basic fibroblast growth factor (bFGF), BDNF, and VEGF [81] and may be associated with the eNOS/BDNF pathway [82].

**Figure 1:**
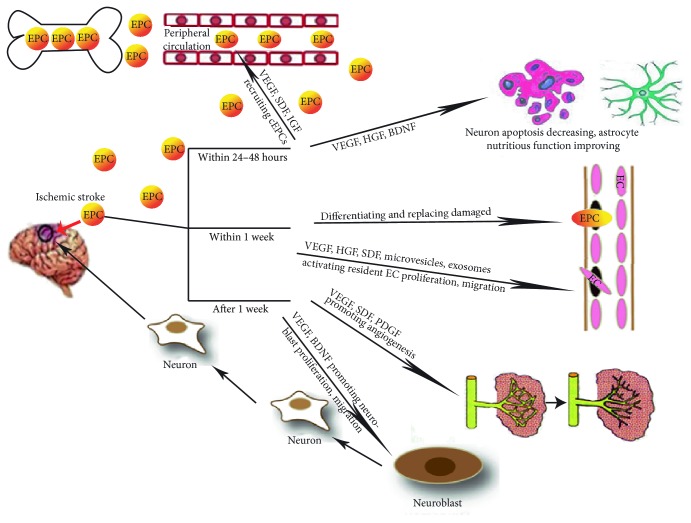
EPCs interact with the neurovascular unit. In the early stage (within 24 to 48 hours), EPCs provide nutritional support for glial cells and reduce neuronal apoptosis through secreting cytokines; during the acute phase (within 1 week), EPCs repair the blood-brain barrier (BBB) and reduce cerebral edema by replacing and repairing the vascular endothelium or promoting the proliferation and migration of resident ECs, thereby reducing nerve cell injury in the ischemic penumbra; in the late acute phase (after 1 week), EPCs recover and reconstruct the neurological functions of nerve cells in the necrotic region by promoting angiogenesis, blood supply, and proliferation and migration of neuroblasts. The figure partly refer to Li et al. [63].

In short, complex interactions between EPCs and the neurovascular unit take place in the ischemic area. In the progress, EPCs and the factors they secrete jointly contribute to poststroke angiogenesis and neurogenesis, reconstructing the functions and structures of vascular and neural networks, which promote the recovery of neurological function after ischemic stroke [78] (Figure 1).

## 3. Application

### 3.1. Clinical Trial

Clinical trials for EPCs used as a marker of prognosis or transplanting therapies have been or are being carried out, primarily targeting the limbs and the cardiovascular and cerebrovascular ischemia. The number of EPCs can be used as a marker of endothelial dysfunction in cardiovascular diseases [83–85]. In the case of acute coronary events or myocardial infarction, the growing number of EPCs indicates that EPC-mediated repair is a physiological response to severe cardiovascular events [86–88]. In the observation of 122 patients with coronary heart disease and normal control group, the number of circulating EPCs was significantly decreased in patients with coronary heart disease [89–91]. Adams et al. verify that mobilization of lin-2/Sca-1+/c/kit + cells into the peripheral blood could be motivated in a long-term treatment of PTH followed by G-CSF administration in mice [92]. PTH treatment mobilizes endothelial stem cells (ESCs)/EPCs from the bone marrow into the peripheral blood in mice of MCAO, which enhances tissue repair and function recovery and reduces adverse immune response [93]. Some trials applied patients' own EPCs mobilized and recruited by G-CSF [94] to the site of myocardial infarction; some used EPCs from the bone marrow in the coronary artery of patients with myocardial infarction [95–97] and successfully recovered the function of the left ventricle; some have started the second phase trials [98–100]; and some trials conducted direct endocardial injection of unfractionated bone marrow cells [101] or injection of mononuclear cells from patients' own bone marrow in critical limb ischemia [102], both of which have improved ischemic symptoms.

It has been proven that the level of circulating EPCs is an independent predictor of the prognosis of patients with acute ischemic stroke [103]. High levels of EPCs in these patients indicate that the infarct volume is smaller and less likely to develop, which may be a marker for the severity of acute stroke [104]. Clinical observational trials have shown that the number of circulating EPCs significantly decreased in patients with cerebrovascular disease than control subjects [105], and the absence of circulating EPCs is associated with increased risk of future vascular events, but not indicating recurrence of stroke [106]. In the ten cases of acute middle cerebral artery infarction, it has been proven to be viable and safe to conduct intravenous injection of patients' own mononuclear cells within 72 hours after onset [107]. Several studies have been conducted or are still undergoing, but with no available results reported, with the purpose of assessing the safety and efficacy of autologous stem cell administration to treat ischemic stroke. Most clinical trials are focusing on bone marrow- or adipose tissue-derived mesenchymal cell transplantation (NCT02378974; NCT01091701; NCT01461720; NCT01678534; NCT01716481; NCT01922908; NCT01297413; NCT00875654; NCT02580019;NCT01714167; NCT02580019; NCT01714167; NCT02580019; and NCT02564328). The remaining studies use peripheral blood- or umbilical cord blood-derived hematopoietic stem cells intracerebrally or infused into the middle cerebral artery of patients (NCT01518231; NCT01249287; NCT00761982; NCT01438593; and NCT00950521) [108]. In this review, we have also queried clinical trials of EPC application in ischemic stroke in ClinicalTrials.gov (Table 1), which have no available results reported.

### 3.2. Time and Methods in Clinical Application

Due to continuous changes in the microenvironment of the stroke site, the timing of stem cell transplantation is a factor that must be considered. However, current animal and clinical trials have not identified a perfect timing for transplantation. Transplantation within 24 hours of stroke has been partially demonstrated to have neuroprotective effects [109, 110]. In some trials, neural stem cell (NSCs) transplantation was used to treat stroke, and it was found that when the transplantation was conducted on the second day after onset, the number of surviving cells was greater compared with the transplantation done in the sixth week [111]. Taking into account the excitotoxicity, brain edema, inflammatory response, and the expression of nutritional factors and other factors, most researchers believe that 7 days after the onset of stroke is a better time for transplantation, because at this time the brain microenvironment has entered the stage of promoting regeneration [112].

Stem cells can be transplanted in the following ways: intracerebral or intracerebroventricular injection, intravascular infusion, and intranasal delivery [113]. Transplanted stem cells may appear in the damaged core and surrounding areas [114]. Different transplantation methods will affect the cell migration, distribution, and number of cells in the target area [115]. In addition, it is also necessary to take into account the type of disease, the dose of transplanted cells, and the timing of transplantation [113]. In the clinical trial in ischemic stroke, intravascular infusion of EPCs, especially super selective injecting into the ischemic area, maybe a feasible and effective approach.

### 3.3. Combination of EPCs and New Technologies

#### 3.3.1. Combination of EPCs and Neurovascular Intervention

The experiment of using EPCs to be implanted on several different scaffolds to form microvascular networks [116] or using stents of collagen-coupled CD34 antibody seeded with EPCs transfected with the A20 gene [117] has become a very promising approach. Blindt et al. has designed an EPC-capturing stent, instead of an EPC-covering stent [118], and a short-term result using such a stent is feasible and effective in a clinical trial [119–121], which is helpful to lead to further development of tissue-engineered stent. Another approach is to design clinical trials, in which intra-arterial EPC perfusion is conducted before or after the intravenous t-PA with mechanical thrombectomy bridging therapy or stent implantation in the intracranial and extracranial artery, so that a high concentration of EPCs is formed; then observe the indicators of postoperative brain edema, vascular reendothelialization, postoperative restenosis rate, and neurological function recovery, so as to find out whether the combination of EPCs transplantation and neurovascular intervention technology is better in protecting the neurovascular unit. In the process, patients of the selective operation implant stent in the intracranial and extracranial artery, and EPCs from the periphery blood or bone marrow are perfused through a hyperselective catheter during the operation. The cell number for implantation is referred to the paper [122, 123]: 20 × 10^6^ or 3 × 10^6^. The clinical trial is not perfect and the detail is not completed now. With the rapid development of neurovascular intervention, the combined application may be a direct and effective way to utilize EPCs and also overcome side effects of the stent treatment and provide expansive prospect in clinical therapy in ischemic stroke.

#### 3.3.2. Combined Transplantation of EPCs and Cytokines

The combined transplantation of FGF-2/PDGF-BB and EPCs has been proven to promote EPC migration [124]. SDF-1*α* and VEGF alone decreased apoptosis, and they may play synergistic role in promoting cell survival and the angiogenesis of EPCs [125]. There is also a study in combined therapy of FGF-2 and G-CSF with EPCs to improve the angiogenic effect in mouse hind limb ischemia models [126]. In the poststroke local acidic environment (pH 6.5), the biological activity of EPCs is impaired, and TPO, stem cell factor (SCF), and IL-3 each could reduce the exposure of EPCs to acid-induced apoptosis. The combined transplantation of the three factors and EPCs can stimulate EPC proliferation and reduce apoptosis, which may be a better choice for vascular endothelial repair and angiogenesis [125]. In the future, growth factor analogues that are more stable in low pH condition may provide better therapeutic strategies with combined transplantation of EPCs.

#### 3.3.3. Combination of EPCs and Synthetic Particles

A nanoparticle is an ideal carrier whose shape, size, surface charge, composition, and coating can be highly customized. It can also protect its carriers and may be released in a controlled manner [127–130]. Nanoparticles can be implanted in molecules, such as VEGF, FGF-2, transforming growth factor-*β* (TGF-*β*), G-CSF, and PDGF [108], that promote EPC function and coated the surface with the amino acid sequence LQNAPRS, which has recently been shown to recognize CD133 [131] and anti-CD34 antibodies that are used to recognize EPC [132], which is a type of nanoparticle that contributes to EPC survival and promote angiogenesis. Experiments were carried out using a synthetic pH-sensitive polymer (urethane spherical sulfamethazine) to load SDF-1*α* and release it in the local acidic environment of the cerebral infarction [133]; other experiments used computer to redesign SDF peptide analogues, which would more effectively induce EPC migration [134] and enhance neurogenesis and angiogenesis. This process may be related to SDF-1*α*/CXCR4 interaction and recruitment of more EPCs, MSCs, and NSCs.

#### 3.3.4. EPC Modification and Pretreatment

To enhance the therapeutic effect, EPCs can also be used for its modification, mainly gene transduction. Experiments have been conducted to use transduced EPCs to overexpress CXCR4, VEGF, IGF-1, hypoxia-inducible factor-1 (HIF-1), eNOS, and other genes, and the transplantation has achieved positive results [135–138]. Other studies used virus-transduced EPCs to overexpress VEGF, which enhanced EPC proliferation and promoted angiogenesis [139]. Compared with conventional EPCs, using EPCs to overexpress anticoagulant and vascular protection genes more effectively reduce pathological vascular remodeling [140, 141]. Due to the fact that stem cells can secret a variety of factors, it is also possible to overexpress antiapoptotic or angiogenic factors through gene manipulation before transplantation, such as kit ligands, VEGF, and FGF2 [142–144]. These gene modification strategies are likely to enhance the therapeutic effect of EPCs [145]. Another method of enhancing the function of EPCs is ischemic preconditioning, which can increase the expression of VEGFR2 on EPCs, thereby promoting the angiogenic effect of EPCs after application [146]. Other preconditioning triggers have been tried out in stem cells or progenitor cells including hypoxia, hydrogen sulfide, hydrogen dioxide, carbon monoxide, and some cytokines and pharmacological agents. The preconditioned stem/progenitor cells show enhanced paracrine effects and better cell survival, which promote functional recovery much better [147]. Alternative test of EPCs is to coincubate with SDF-1*α*, which has also promoted angiogenesis in the limb ischemia models [148] (Table 2). These studies suggest that enhancing the EPC function through modification techniques and pretreatment may have a greater advantage in the treatment of ischemic stroke.

### 3.4. Safety

The safety and potential risks of EPC transplantation are also validated in some studies. The impact of EPC effect on formation and progression of atherosclerotic plaques still remains controversial [149], which may be involved with a more accurate phenotypic characterization of EPCs [145]. It has been found that bone marrow-derived EPCs are associated with early angiogenesis in tumors, and in later tumors, these neovessels are diluted by vessels from the periphery [150], which indicate that EPCs are involved in the earliest phases of tumor angiogenesis and therefore EPCs transplantation should not be applied to tumor patients [78]. EPCs may also increase ischemia-induced inflammatory factors, including IL8, monocyte chemotactic protein-1 (MCP-1), and recruit mononuclear-macrophages, thereby aggravating ischemic injury [53, 151, 152]. After EPC transplantation, the connection between nascent capillary endothelial cells is not tight enough and the permeability is high, which may aggravate brain edema [153] and increase the risk of bleeding. EPCs and paracrine VEGF promote angiogenesis, which may lead to uncontrolled growth of local capillaries, developing into hemangioma or capillary groups. Other possible side effects include epilepsy, direct injection-induced injury, and transplantation failure caused by allotransplantation-induced immune responses [154, 155]. In the current clinical trials, there are some limitations which include lack of appropriate controls, randomization, blinding, and a small number of patients followed up for short periods [145]. However, transplantation of EPCs in patients with acute myocardial infarction did not affect plasma C-reactive protein and leukocyte levels [96] and did not lead to tumor angiogenesis in the 5-year follow-up [99]. More experimental animal studies of EPC-based therapy, especially in ischemic cerebrovascular disease, and systemic designed clinical trials should be carried out to interpret the safety issues of EPC application in the future.

## 4. Conclusion

As a kind of adult stem cells, EPCs' biological characteristics have been determined to repair BBB, improve microcirculation, reduce neuronal apoptosis, and promote the proliferation and migration of neural stem cells through replacing and repairing vascular endothelial cells, promoting angiogenesis, and secreting cytokines and growth factors, which have enabled it to protect the neurological vascular unit. The combination of EPC transplantation with neurovascular intervention, synthetic particles, gene modification, and other technologies will further enhance the therapeutic effect of EPCs and play a more significant role in the treatment of ischemic stroke. There may be a promising approach of EPC application although some safety issues need to be solved.

## Figures and Tables

**Table 1:** Clinical trials for ischemic stroke with endothelial progenitor cells.

References	Study type	Estimated enrollment	Recruitment status	Start date	Investigator
NCT01289795	Observational	30	Unknown status	2010.7	Matthias Endres
NCT01468064	Interventional	20	Recruiting	2011.11	Zhenzhou Chen
NCT02157896	Observational	30	Completed	2013.5	Hao Chen
NCT02605707	Interventional	30	Recruiting	2014.11	ZhenZhou Chen
NCT02980354	Observational	200	Recruiting	2017.2	Ulvi Bayraktutan

**Table 2:** Combination of EPCs and cytokines or pretreatment.

Cytokine or pretreatment	Approach	Effect
FGF-2/PDGF-BB	Combined transplantation	EPC migration ↑
SDF-1*α*/VEGF	Combined transplantation	EPC apoptosis ↓
SDF-1*α* + VEGF	Combined transplantation	EPC survival ↑, angiogenesis ↑
FGF-2/G-CSF	Combined transplantation	Angiogenesis ↑
TPO + SCF + IL-3	Combined transplantation	EPC proliferation ↑, apoptosis ↓
SDF-1*α*	Coincubation	Angiogenesis ↑
Nanoparticle	Carrying cytokines	EPC function ↑
Gene transduction	Overexpressing cytokines	EPC function ↑
Ischemic preconditioning	Increasing VEGFR2 expression	Angiogenesis ↑
